# Aquatic Food Products: Processing Technology and Quality Control

**DOI:** 10.3390/foods13172806

**Published:** 2024-09-03

**Authors:** Jingran Bi

**Affiliations:** State Key Laboratory of Marine Food Processing & Safety Control, Dalian Polytechnic University, Dalian 116034, China; bijingran1225@foxmail.com; Tel.: +86-0411-86322020

## 1. Introduction

Aquatic products have the characteristics of high protein, low fat, and good nutritional balance, and they have become an important source of support to solve world hunger and nutritional deficiencies. More than 900 million people around the world consume most of their daily protein through aquatic products [1], which are gradually becoming an important part of human diet: aquatic product-derived protein intake accounts for 2% of the total animal protein intake per capita [2]. Aquatic products also are an important source of polyunsaturated fatty acids, which have important functions in human health: providing heat, being a carrier of fat-soluble substances, protecting heart health, clearing excess lipids from blood vessels, regulating immune metabolism, etc. [3,4]. In addition, aquatic products have a delicious taste, which is widely welcomed by consumers.

The perishability of aquatic products makes them susceptible to bacterial spoilage throughout the fishing, transportation, processing, and storage stages. Following the spoilage of aquatic products, enzymatic activity within them facilitates the decomposition and degradation of nutrients, leading to the formation of malodorous and toxic substances [5]. The quality and safety of aquatic products are often measured by K value (representing the ratio between the sum of inosine and hypoxanthine and the sum of all other adenosine triphosphate breakdown products), volatile basic nitrogen, biological amine, and other biochemical indexes [6]. Spoilage greatly reduces the edible value and economic value of aquatic products, resulting in a great waste of resources. Therefore, improving the preservation technology of aquatic products and reducing their spoilage rates is one of the problems that the aquatic product processing industry needs to solve. Traditional freshness preservation methods are mainly chemical preservation, including salt, acid, smoke, etc. [7]. Chemical preservation methods, although effective in inhibiting bacterial growth and despite boasting a high level of sterilization efficiency, may potentially compromise the texture, distinctive umami quality, and delicate taste of aquatic products. Additionally, they may leave behind chemical residues, contributing to environmental pollution. With the increasing demand for food safety and health, some chemical and physical preservation methods that destroy nutrients are rejected by consumers. In recent years, extracting natural substances from plants to ensure the quality and safety of food has become a research trend. In addition, it is worth nothing that bacteria are the most important factor causing the deterioration of aquatic products, as they can gradually decompose large molecules such as proteins and lipids in aquatic products into small molecular compounds such as amino acids and fatty acids, leading to the generation of harmful substances such as amines and hydrogen sulfide and to the production of unpleasant odors [8,9]. Currently, as the scholarly exploration into bacterial quorum sensing (QS) systems intensifies, the pivotal role bacterial QS plays in the degradation of aquatic products has been gradually discovered. Researchers have strived to elucidate the molecular mechanisms of the effects of QS signaling molecules on nutrient degradation [10]. As QS can inhibit or slow down the process of aquatic product spoilage, it has provided a new way of aquatic product preservation.

Proper processing can prevent spoilage and extend the shelf life of aquatic products while maintaining their nutritional value, texture, and flavor. At present, the commonly used processing methods of aquatic products include hot processing, pickling, high-pressure and ultrasonic methods, and so on [11]. Heating is the most common way of processing aquatic products, and it can easily change the flavor of food and its nutritional composition. The traditional heating method uses radiation to heat the surface of an object and then gradually heats the interior through conduction and convection [12]. Using this method during processing can involve problems such as low heating efficiency, long local heating time, short internal heating time, uneven heating, and others, which leads to the loss of nutrients, bad taste, and a dry, rough texture of aquatic products. In addition to the fresh consumption of aquatic products, curing is also a favorite processing method; it can not only inhibit the multiplication of microorganisms, but also extend the preservation time of aquatic products [13]. High-pressure treatment is a non-heat treatment process that can replace heat treatment. Ultra-high-pressure processing is not affected by the size and shape of aquatic products, because the pressure applied during processing is uniform. Ultra-high-pressure processing can inactivate microorganisms at lower temperatures, ensuring the safety of food and extending its shelf life. Unlike hot processing, ultra-high-pressure processing can break down non-covalent bonds in proteins, squeezing them and changing their molecular conformation, but it has no effect on the covalent bonds of small molecular substances such as amino acids, so it can maintain food flavor and nutrition to a large extent [14,15]. As an advanced food processing technology, ultrasonic processing has broad prospects in food processing and preservation. It is a mild but targeted form of processing that can improve the quality and safety of aquatic products. Although ultrasound treatment alone is not sufficient to inactivate the various harmful enzymes in aquatic products, ultrasound has shown potential for a highly efficient inactivation of enzymes and pathogens compared to mild heat treatment [16]. As an important nutrient component of aquatic products, protein plays an important role in aquatic product quality during processing. The spatial structure of proteins is maintained by secondary bonds (such as hydrogen bonds), which change from their original ordered spatial structure to a disordered spatial structure under the various processing methods [17]. Changes in protein structure can affect the properties of protein in the following ways: (i) physicochemical properties: the tight structure becomes loose, the hydrophobic groups are exposed, the asymmetry of protein increases, and the physicochemical indexes (such as solubility) change [18,19,20]; (ii) biological activities: function is changed; (iii) biochemical properties: the protein denatures and the crystal structure disappears and can be easily hydrolyzed by protease. The application of diverse processing methods leads to varying degrees of protein alterations, consequently affecting the flavor of aquatic products and their nutritional composition [18,19,20]. Therefore, it is of great significance to study the influence of aquatic product processing methods on protein properties for the development of superior processing technologies.

This Editorial refers to the Special Issue “Recent Advances in Aquatic Food Products Processing”. The Special Issue, which contains nine research articles and one review, highlights traditional processing (such as heating, salting, drying, smoking, natural fermentation), modern processing (such as ultra-high-pressure processing, low-salt fermentation, rapid freezing-thawing, etc.), and byproduct processing, as well as the quality change mechanisms that occur during the processing of aquatic products. To encourage readers to explore this Special Issue, I briefly describe the various contributions in the following paragraphs.

## 2. An Overview of Published Articles

The paper by Hematyar et al. (contribution 1) reported the relationship between fish welfare and ultimate filet quality. The study evaluated the effects of pre-slaughter handling and stocking density on filet quality in largemouth bass (*Micropterus salmoides*). The fish were divided into three groups and reared at various stocking densities of 35, 50, and 65 kg·m^−3^, respectively. After feeding for 12 weeks, half of the fish were slaughtered by direct percussion on the head without air exposure (control group), and the other half were subjected to acute antemortem stress through anoxia for 3 min. Blood and filet samples were collected for quality and nutritional evaluation. It was found that anoxia triggered an earlier occurrence of rigor mortis, with a lower initial postmortem pH than that of the control group. Moreover, the stocking density and anoxia might have caused stress in the fish. When the fish were stimulated, their blood cortisol and plasma glucose levels climbed dramatically. Moreover, the stressful rearing environment at high stocking density also contributed to the hypoxia, resulting in the rapid depletion of glycogen reserves and the antioxidant defense. The oxidative enzyme activities in the intensive stocking groups were significantly higher than those of the sparse stocking group. Furthermore, low stocking density and pre-slaughter handling without anoxia also exhibited a slowing effect on the lipid oxidation and protein oxidation process by metal catalyzation, which was beneficial to the textural parameters of filets. In conclusion, fish welfare plays an essential role in the improvement of filet quality.

Aussanasuwannakul et al. (contribution 2) revealed crucial elements of online market success for traditional fermented aquatic foods. In detail, this work evaluated the nutritional composition, storage stability, sensory quality, and packaging efficiency of roasted pickled fish powder (RPFP), underlining the balance between the characteristics of traditional food and consumers’ health requests. Based on the commercial benchmark, the protein content was measured at 40.17%, and the fat content was 10.60%. Compared to the commercial benchmark, the developed RPFP exhibited a higher protein content in the herbal flavor product, with a value of 28.97%, and a superior fat content in the spicy flavor products, at 19.51%. Moreover, the herbal flavor product was rich in dietary fiber, at 14.23%, and the intense heat of the spicy flavor product could effectively comply with both the nutritional and specific taste expectations of customers. Moreover, through storage stability analysis, including the determination of microbiological, pH level, and water activities, packaging forms such as traditional polypropylene cups and laminated aluminum foil stand-up pouches were evaluated. It was demonstrated that packaging efficiency could not only extend the shelf life of products thanks to excellent barrier properties, but also improve brand performance. Furthermore, the novel method of estimating both the nutritional and sensory dimensions of RPFP products for online market success provided invaluable insights into enhancing the status of traditional food in the field of e-commerce.

Wang et al. (contribution 3) focused on the regulation of quorum sensing (QS) in the microbial coculture system. *Hafnia alvei* (*H. alvei*) and *Pseudomonas fluorescens* (*P. fluorescens*) are the spoilage microorganisms specific to aquatic foods. The authors found that the biofilm contents, extracellular polysaccharides, and biogenic amines in the coculture system of *H. alvei* and *P. fluorescens* were significantly decreased as the *luxI* gene of *H. alvei* was knocked out, implying that QS participated in the dual-species interactions. Moreover, transcriptomics was applied to illustrate the regulatory mechanisms at the molecular biological level. In the coculture system of *H. alvei* ∆luxI and *P. fluorescens*, the transcriptional levels of genes associated with chemotaxis, flagellar assembly, and two-component system pathways were dramatically down-regulated. In addition, in the coculture system, 732 of the genes identified in *P. fluorescens*, mainly related to biofilm formation, ATP-binding cassette transporters, and amino acid metabolism, were significantly differentially expressed. These results demonstrated that the disruption of QS in *H. alvei* could effectively weaken spoilage development in the microbial coculture system, which offers a new insight into the inhibition of *H. alvei* and *P. fluorescens* and into the preservation of aquatic products.

Zhang et al. (contribution 4) developed a rapid non-contact cutting position identification method based on a constructed linear laser data acquisition system. This method eliminated the reliance on manual subjective experience, consequently making it suitable for large-scale production lines. The 3D point cloud information of the surface contour of the fish body was nondestructively collected via line laser scanning technology. Using principal component analysis (PCA), the characteristic variables of the ventrodorsal boundary line were extracted, which reduced the time-consuming computation and enhanced the recognition ability to identify the cutting position. Then, the fish head identification models were established using Least Squares Support Vector Machines (LS-SVMs), Particle Swarm Optimization-Back Propagation (PSO-BP) networks, and Long and Short-Term Memory (LSTM) neural networks, respectively. The paper found that the LSTM model was superior, with minimal error between the predicted and actual values and with satisfactory reliability. Further, from the viewpoint of fish morphology, this identification technology based on the ventral-dorsal demarcation line is reasonable and therefore has strong potential in the development of intelligent fish processing and equipment to meet the decapitation needs of salmon and tuna and other large carp-shaped fish.

Li et al. (contribution 5) proposed an amino-carboxymethyl chitosan (ACC)/dialdehyde starch (DAS) film to effectively load and slow-release ε-polylysine (ε-PL), which could be applied in the aquatic preservation field. The content of amino groups on chitosan was 0.83 ± 0.02 mmol/g after modification by carboxymethylation and amination, and the aldehyde content of starch was 89.8 ± 0.07% after oxidation by periodate. The ACC/DAS film was prepared by the Schiff base reaction between the amino group on ACC and the aldehyde group on DAS, which built a firm three-dimensional network skeleton structure. Due to occurrence of the Schiff base reaction, imide bonds (-NCH-) were formed, and the two long-chain polysaccharides were cross-linked, thus disrupting the crystal arrangement. Swelling analysis found that the ACC/DAS porous structure had a strong water-holding capacity. However, the over cross-linking network prepared by ACC/DAS with a -NH2: CHO molar ratio of 1:0.6 displayed a low flexibility, which restricted the penetration of water molecules. The loading ratio of ε-PL on ACC/DAS was 99.2%. Combined with the antibacterial activities of ε-PL and ACC, ACC/DAS/ε-PL exhibited an excellent long-time inhibition capability against *S. aureus* and *E. coli*, which makes it a good candidate for active food packaging.

Li et al. (contribution 6) explored the food safety of shrimp paste fermented by a new starter culture, *Tetragenococcus muriaticus* TS (*T. muriaticus* TS). Shrimp paste, rich in nutrients and with a unique flavor, is popular in Southeast Asian and Chinese coastal areas. During the traditional fermentation process of shrimp paste, biogenic amines, which are a hazardous food factor, are always generated by microorganisms via the decarboxylated reduction of amino acids. The autochthonic salt-tolerant *T. muriaticus* TS exhibited a positive amine oxidase activity, which could degrade biogenic amines in vitro. According to high-throughput sequencing data, *T. muriaticus* TS could disrupt the bacterial structure and interspecific correlations, and especially suppress the growth of bioamine-producing bacteria. Consequently, after fermenting for 35 days, putrescine, cadaverine, and histamine concentrations in shrimp paste were significantly reduced by 19.20%, 14.01%, and 28.62%, respectively. Meanwhile, *T. muriaticus* TS also effectively inhibited the total volatile base nitrogen and improved the amino acid nitrogen concentrations. Through HS-SPME-GC-MS analysis, it was found that the odor of the shrimp paste fermented by *T. muriaticus* TS was improved, and pyrazines were enhanced while amines were weakened. Overall, *T. muriaticus* TS is an effective starter culture to prepare shrimp paste with high flavor quality and safety.

Zhu et al. (contribution 7) studied the influence of drying methods on pufferfish filet quality. Hot air drying (HAD), a common aquatic food product processing method, always effectively improves the drying speed. Cold air drying (CAD) is an appropriate approach to retain sensory quality and nutrition. Cold and hot air combined drying (CHACD) may combine the advantages of the two and avoid their disadvantages. The study found that during drying, mobile water molecules were reduced via evaporation, and the immobilized water content of CHACD was between that of HAD and CAD. CHACD also enhanced the textural attributes of the filets, such as their springiness, chewiness, and toughness, owing to their intact, well-arranged, and well-defined microstructure. Although drying inevitably causes lipid oxidation and protein degradation, CHACD could effectively alleviate this problem. All in all, CHACD exhibits advantages over CAD and HAD, such as an accelerated drying time, reduced lipid oxidation, improved protein stability, and tightened tissue structure, providing a novel drying method for pufferfish processing.

Qian et al. (contribution 8) worked on modified atmosphere packaging (MAP), which is an eco-friendly and convenient physical technique to prolong the shelf life of food. In their paper, the effects of MAP’s gas composition, including carbon dioxide (CO_2_), nitrogen (N_2_), and oxygen (O_2_), on the quality of salmon were studied. The study found that MAP with a CO_2_ concentration of over 40% could effectively inhibit the growth of mesophilic bacteria. However, excessive CO_2_ (≥80%) might facilitate proteolysis, thus providing nutrients for bacterial growth, resulting in an increase in the total mesophilic bacterial count and the accumulation of TVB-N, as well as in a decrease in hardness, water-holding capacity, and myofibrillar protein content. Furthermore, a MAP environment with an O_2 content_ higher than 20% probably promotes protein degradation, consequently affecting muscle texture. According to electronic nose analysis, 60% CO_2_/20% O_2_/20% N_2_ and 60% CO_2_/10% O_2_/30% N_2_ were most suitable to retain the original odor characteristics of salmon. In summary, 60% CO_2_/10% O_2_/30% N_2_ was the optimal MAP gas composition for the preservation of salmon by inhibiting bacteria growth and resisting protein degradation.

Chen et al. (contribution 9) described the processing quality of crabs during thermal processing. Along with thermal processing, protein degradation is closely related to the nutrition, texture, and flavor profiles of aquatic products. For example, free amino acids (FAAs) are the end products of protein degradation that provide the umami, sweet, and bitter tastes of heated aquatic foods. It was found that bitter amino acid content in crabs did not undergo any significant change during steaming, while sweet (Thr, Gly, Ala, and Pro) and umami amino acids (Asp and Glu) climbed to the maximum value after 10 min of steaming and then declined. Furthermore, flavor nucleotides such as guanosine monophosphate, inosine monophosphate, and adenosine monophosphate also exhibited a synergistic effect with free amino acids on the umami and sweetness profiles of crabs during steaming. Combined with electronic tongue analysis and sensory assessment, the recommended steaming time for crab was found to be 10–15 min. These findings provide a theoretical foundation for the large-scale standardized processing of crabs.

Finally, Fan et al. (contribution 10) reviewed the characteristics, mechanism, and inhibition of endogenous proteases in sea cucumber (*Apostichopus japonicas*). The autolysis of sea cucumber is always triggered by ultraviolet irradiation, mechanical injury, ambient temperature, etc. During autolysis, endogenous proteases play an essential role in damaging collagen fibers or the interfibrillar proteoglycan bridge, resulting in tissue self-dissolution, which causes a decrease in edible suitability and economic loss. Therefore, it is necessary to systematically understand the action mechanism and inhibition methods of endogenous proteases. In the paper, the external trigger factors of sea cucumber autolysis and the changes in protein composition and mechanical properties during autolysis were described in detail. Furthermore, the classification, characteristics, possible mechanisms, and natural inhibitors of endogenous proteases were specifically reviewed. Additionally, it was pointed out that molecular biological mechanisms, optimization processes, and applications of effective protease inhibitors in the sustainable production of sea cucumber should be the focus of future research.

## 3. Conclusions

This compilation of articles devoted to describing the sensory quality, protein degradation, and hazard generation and inhibition in the process of aquatic product storage, preservation, fermentation, and hot processing provides a theoretical basis for the growth of the aquatic product industry.

With the continuous development of the global aquatic product market, the improvement of aquatic product processing technology has become the main research direction. The current processing technology of aquatic products mainly includes defishment, prefabrication, sterilization, and preservation, etc. These processing technologies can effectively improve the quality of aquatic products, improve their taste, extend their shelf life, and maintain their nutritional value. Meanwhile, the focus of research on aquatic product processing technology has gradually shifted from a single method to a multi-technology composite application to better preserve the nutrients in and taste of food. The quality improvement process of aquatic products needs to integrate a variety of high-tech processing technologies, and the exploration, development, and large-scale application of new processing technologies are the necessary conditions for its future development.
